# An Amperometric Immunosensor Based on a Polyelectrolyte/ Gold Magnetic Nanoparticle Supramolecular Assembly—Modified Electrode for the Determination of HIV p24 in Serum 

**DOI:** 10.3390/molecules15075053

**Published:** 2010-07-23

**Authors:** Ning Gan, Jianguo Hou, Futao Hu, Lei Zheng, Minjun Ni, Yuting Cao

**Affiliations:** 1 State Key Laboratory Base of Novel Functional Materials and Preparation science, Faculty of Material Science and Chemical Engineering, Ninbo University, Ningbo, 315211, China; 2 Hospital, Southern Medical University, Guangzhou, 510515, China

**Keywords:** electrochemical biosensor, polyelectrolyte/gold magentic nanoparticle, immunosensor, HIV p24

## Abstract

A novel supramolecular amperometric immunosensor for the determination of Human Immunodeficiency Virus antigen p24 (HIV p24) was built up using the electrostatic layer-by-layer self-assembly technique upon a gold electrode with HIV p24 antibody (anti-p24) being immobilized on polyelectrolyte/gold nanoparticle multilayer films. The multilayer films were composed of poly(L-lysine) (pLys) and mercaptosuccinic acid (MSA) stabilized Fe_3_O_4_(core)/gold(shell) nano particles (GMPs).The immunosensor preparation steps were monitored by X-ray fluorescence spectrometry (XRFS), scanning electron microscopy (SEM) and transmission electron microscopy (TEM). In pH 6.5 PBS, after the immunosensor was incubated with HIV p24 solution at 25 °C for 5 min, the electron transfer access of FeCN is partially inhibited, which leads to a linear decrease of peak current. In addition, the performance of the immunosensor was studied in detail. It offers high-sensitivity for the detection of p24 and has good correlation for the detection of p24 in the range of 0.1 to 100.0 ng/mL with a detection limit of 0.05 ng/mL estimated at a signal-to-noise ratio of 3. The proposed immunosensor was used to analyze p24 in human serum specimens and the results showed the developed immunosensor provides a promising alternative approach for detecting p24 in the early diagnosis of AIDS patients.

## 1. Introduction

There are currently an estimated 700,000 people living with HIV in China, including about 75,000 AIDS patients [1]. There is currently no curative therapy available for acquired immunodeficiency syndrome (AIDs), so detecting HIV at an early stage is the best hope of decreasing the mortality rate [2]. Some procedures for the detection of HIV relying on immunoassay recognition of HIV antibody (anti HIV) are available, but in the early stages of HIV infection when anti HIV has not yet appeared in the sufferers’ serum (the window period), the patient is highly infectious [3]. If detection during this period is attempted, it is bound to miss patients suffering from HIV. Nevertheless, one of the diagnostic markers of HIV glycoprotein antigen (p24) has been found in human serum in the "window period", although its content is very low (<5 ng/mL) in the early one week after infection [4]. If p24 is found at this time, HIV can be diagnosed, which will effectively shorten the “window period ". At present, enzyme-linked immunoassay assay (ELISA) relying on a change in color detected by fluorescence, luminescence or radioactive emission is the main method to detect trace amounts of p24 [5]. However, these conventional immunoassay methods need enzyme or fluorescent-labeled antibody/antigen, not to mention its lengthy analysis that requires highly skilled personnel, specially equipped laboratories, and expensive chemicals [6]. Therefore, would be highly desirable for disease diagnosis to develop new methods for fast and convenient monitoring of p24. 

The electrochemical immunosensor is one of the more attractive analytical tools due to several advantages such as specificity, simplicity, direct detection and time savings of the analyses compared with conventional immunoassay techniques [7,8]. As for the construction of an electrochemical immunosensor, the crucial step is the immobilization of an immune probe such as an antibody or antigen onto the electrode surface. One of the traditional shortcomings in the practical applications of electrochemical immunosensor has been the lack of simple methods to fix probes on the electrode surface and ensure their long-term biological activity. Furthermore, the main disadvantages of many reported amperometric immunosensor are the necessity to separate free from bound label which requires washing and separation steps, which increase the complexity of the assays [9]. Furthermore usually an electrochemical transducer molecular (eT) must be added to the reaction system to accelerate electron transfer between the electrode and immunoassay system [10]. In recent years, nano- metal particles have been used as a basic interface to construct antibody protein monolayer [11]. Especially, nonmaterial composed of two- and three-dimensional assemblies of nanoparticles (NPs) with narrow size distribution are becoming increasingly important in analytical and materials chemistry, due to their practical applications in nanoelectronic and optoelectronic devices and biosensors [12]. Using the electrostatic and covalent interactions of bifunctional groups on the substrates, the assembly of individual NPs into three-dimensional structures has become an important and widespread research subject [13,14,15,16,17,18,19,20,21,22,23]. Electrodes modified with gold nanoparticles (AuNPs) are usually fabricated by assembling AuNPs on the electrode surface using organic linker molecules such as thiols and polymers which can provide antibody proteins with a stable environment similar to the native one and thus help retain their bioactivity [14], however the immobilization of AuNPs is usually difficult to achieve. Recently, increasing research has been focused on layer-by-layer (LBL) self-assembly methods using sequential adsorptions of ionized polyelectrolyte and oppositely charged materials in aqueous solutions which has many advantages, such as a being a simple, low temperature deposition, with no limit of thickness and thickness control on the nanoscale [15,18,19,20]. Fe_3_O_4_(core)/gold(shell) composite nanoparticles (GMPs) has been investigated intensively in recent years because of their remarkable electrical and magnetic properties which can be employed for the development of biosensors via magnetic control with an external magnet [16,17]. Anti-p24 can be easily absorbed on GMPs from the solution mixtures with the aid of Au colloids in the outside shell of GMPs to form GMPs-anti-p24 composite probes. Such probes can then be easily separated from free anti-p24 by adding a magnetic field for the super-paramagnetic Fe_3_O_4_. Moreover in GMPs, there are several AuNPs coated on one Fe_3_O_4 _particle, which means that there would be more antibodies immobilized sites than in separate AuNPs.

The focus of this report is the fabrication of a magnetic-controlled electrochemical immunoassay system based on a home-made detection cell for the measurement of p24 with switching and controlling of electrochemical signals by means of an external magnet. The immunosensor electrode was prepared on layer-by-layer self-assembled films which were composed of positively charged poly (L-lysine) (pLys) [19] and negatively charged mercaptosuccinic acid stabilized GMPs(MSA-pLys-GMPs) with an average diameter of 100 nm. We employed GMPs to stabilize HIV p24 antibodies because it has both advantages in magnetic separation and the ability to immobilize antibody proteins with Au colloid. Thus the immunosensor electrode modified with GMPs-anti-p24 would be expected to have a wider detection range than AuNPs modified electrodes. We immobilized MSA stabilized GMPs-anti-p24 in the pLys (pLys/GMPs-anti-p24) membrane layer by layer on the surface of Au|MSA electrode to prepare a p24 biosensor (Au|MSA/{pLys/GMPs-anti-p24}_n_), which was then successfully employed in the detection of trace amounts of p24 in human serum. 

## 2. Results and Discussion

### 2.1. Characterization of the HIV immunosensor

**Figure 1:**
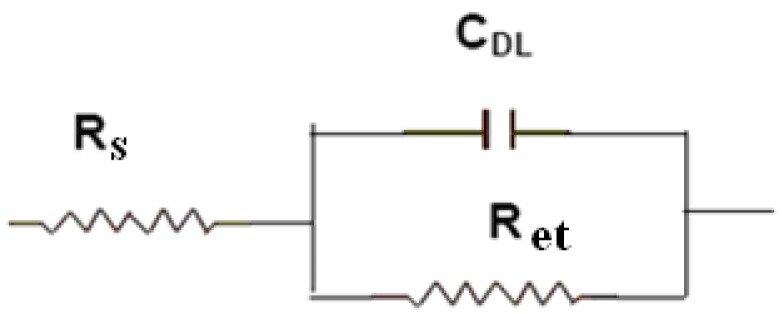
Equivalent circuit used for analyzing the impedance spectra.

**Figure 2:**
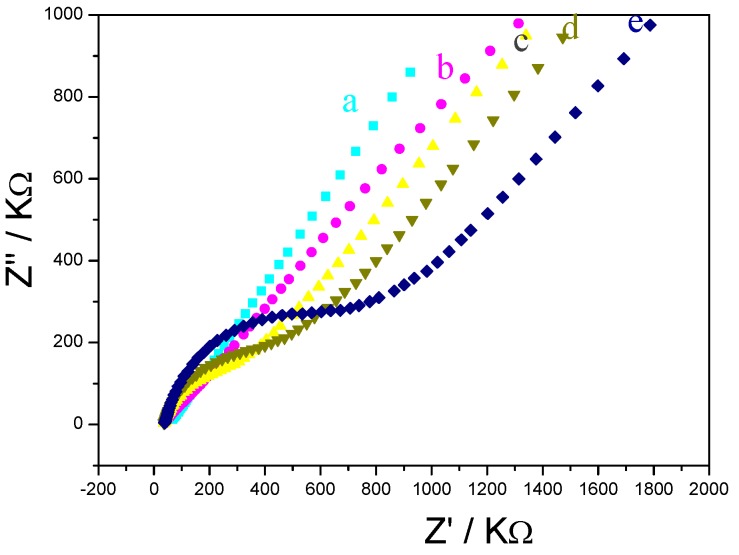
Electrochemical impendance spectroscopy (EIS) of difference electrodes: (a) bare Au electrode, (b) Au|MSA, (c) Au|MSA/{pLys/GMPs-anti-p24} (d) Au|MSA/{pLys/GMPs-anti-p24}_2_，(e)Au|MSA/{pLys/GMPs-anti-p24}_3_ in the presence of 0.1 mol/L KCl and 5.0 mmol/L Fe(CN)_6_^3/4-^; frequency: 1.0 × 10^-2^~1.0 × 10^5^ Hz.

Electrochemical impedance spectroscopy (EIS) has been proven to be one of the most powerful tools for probing the features of surface-modified electrodes [8,20]. The equivalent circuit in Figure 1 has been shown to account satisfactorily for the impedance spectra used here to analyze the pLys/GMPs multilayer. In Figure 1, the solution resistance is represented as *R*s, the double-layer capacitance as C_DL _and the semicircular part at higher frequencies corresponds to the electron transfer resistance (R_et_). Figure 2 shows the electrochemical impedance of the bare Au electrode(a) and the layer by layer immunosensor(b~e) in the presence 5.0 mmol/L K_3_Fe(CN)_6_+K_4_Fe(CN)_6_([Fe(CN)_6_]^3-/4-^). It can be seen that the bare gold electrode exhibited an almost straight line (Figure 2a), which was characteristic of a diffusion limiting step of the electrochemical process [23] can be seen that the change of electrochemical impedance occurred after MSA was modified on the surface of gold electrode (Figure 2b), indicating that the MSA film obstructed electron-transfer of the electrochemical probe. When the positively charged pLys/GMPs-anti-p24 was absorbed on negatively charged Au|MSA, the interfacial resistance increased highly (Figure 2c). It can also be seen that as the number of layers (pLys/GMPs-anti-p24) reached to 2 and 3, the resistance increased(Figure 2d,e) which implies that the deposited film obstructs the electron transfer of the electrochemical probe**.** pLys is positively charged in pH 6.5 PBS buffer and can be easily absorbed by the negatively charged GMPs-anti-p24 as a result of the adsorption of MSA in the fabrication process to obtain the desired number of { pLys/GMPs-anti-p24}_n_ multilayer films.

**Figure 3:**
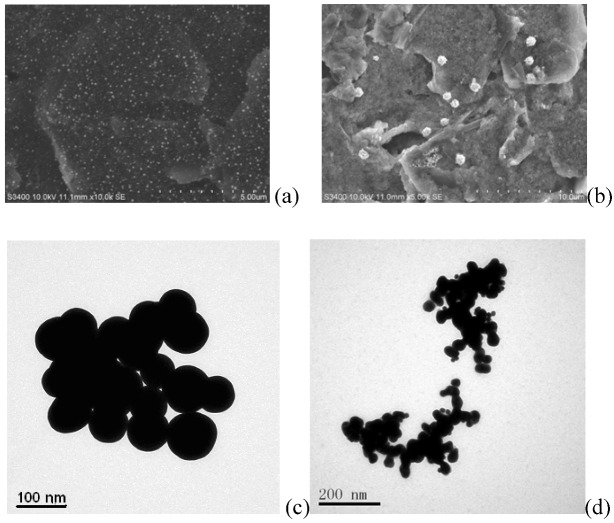
SEM images of the gold electrode coated with (a) Au|MSA/{pLys/GMPs}_2 _films and (b) Au|MSA/{pLys/GMPs-anti-p24}_2_ films. the TEM images of (c) pLys/GMPs, and (d)pLys/GMPs-anti-p24.

The electrode surfaces of (Au|MSA/{pLys/GMPs}_2 _and Au|MSA/{pLys/ GMPs-anti-p24 }_2_) were characterized using SEM (Figure 3). From Figure 3a, one can see that there are many white bright spot on the electrode surface which may be caused by gold colloids on the surface of GMPs that reflect light [16]. The dimension of most GMPs particles was about 100~120 nm. The GMP film can be found evenly on the Au electrode modified with pLys which has a larger surface compared with the ordinary bare electrode. When the anti-p24 was coated on GMPs with aid of pLys to form pLys/GMPs-anti-p24 composite and immobilized on a Au electrode by MSA, the electrode surface morphology had changed markedly. There were a lot of island-based spheres and presumably this was the morphology after the antibody was fixed on the electrode surface (Figure 3b). TEM is an effective method to provide information on particle size and shape. Here, TEM was used to characterize the microstructure of pLys/GMPs (Figure 3c), and pLys/ GMPs-anti-p24 composite (Figure 3d). The average size of pLys/GMPs was about 120 nm, which is slight larger than bare GMPs. As shown in Figure 3d, the pLys/GMPs-anti-p24 composite shows a uniform structure with an average size about 300 nm, which proved that GMPs is an excellent material to immobilize p24 antibodyies because of the virtues of the nano-Au surface to immobilize antibody proteins.

X-ray fluorescence spectrometry (the elements of ^9^F~^92^U can be determined) of Au|MSA/{pLys/GMPs}_3_-anti-p24 showed there were peaks of Fe-kα (2θ = 123.7°) and S-kα (2θ = 110.5°), because p24 is rich in methionine which contains the element S, indicating that GMPs-anti-p24 had been immobilized on the electrode surface. The XPS spectrum of Au|MSA/{pLys/GMPs}_3_-anti-p24 showed sharper and higher peaks corresponding to Au(2P_3/2_, 2P_1/2_)(932.7/954.3 eV) than Au|MSA, which proved the concentration of Au nanoparticles is higher than Au|MSA on the gold electrode surface.

To further investigate the synthesis of the bionanoparticles, FT-IR was used (data not shown). The absorption bands of anti p24 were observed at 1,655 and 1,534 cm^-1^, which were attributed to the protein amide I and amide II infrared absorbance bands. When anti-p24 molecules were modified on the GMP nanoparticle surface, the absorption bands for amide I and amide II were located at 1,674 and 1,560 cm^-1^, respectively, which indicated that anti-p24 immobilized on the surface of the GMP nanoparticles retained their native structure. Moreover, the slight change in absorption wave numbers suggested an interaction between the GMPs and anti-p24. 

### 2.2. Performance of the immunosensor

#### 2.2.1. Cyclic voltammetric behaviors of the immunosensor

**Figure 4:**
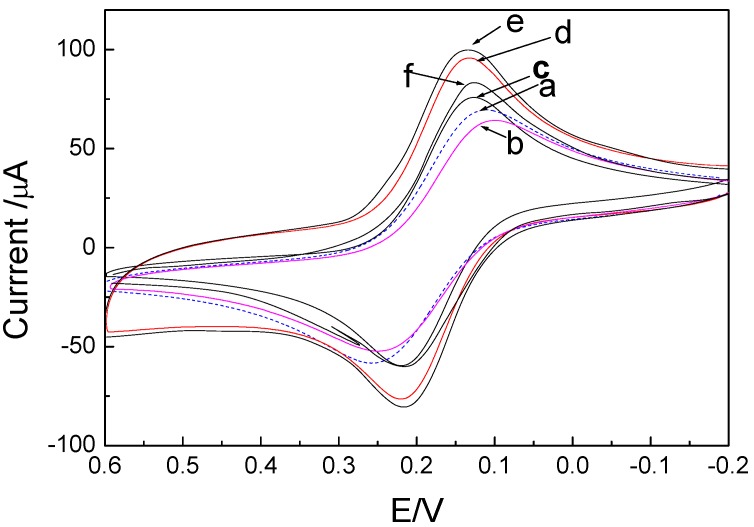
Cyclic voltammograms of the electrode at difference stages: (a) bare Au, (b) Au|MSA, (c) Au|MSA/{pLys/GMP-anti-p24}, (d) Au|MSA/{pLys-GMPs-anti-p24}_2_, (e)Au| MSA/{pLys-nanoAu-anti-p24}_3_, (f) the immunosensor of (d) after incubated in the solution containing 50 ng/mL p24 for 30 min at 37 °C. Supporting electrolyte: 5 mmol/L FeCN(K_3_Fe(CN)_6_) solution containing 0.1 mol/L KCl; scan rate: 100 mV/s. The bare electrode curve (dashed line, n = 0) is shown for comparison.

The cyclic voltammetry (CV) curves of the immunosensor in pH 6.5 PBS are shown in Figure 4. A pair of stable and well-defined redox peak can be observed in Figure 4a. The anodic and cathodic peak potentials were at 215 and 135 mV at 100 mV/s scan rate, respectively, in correspondence with the redox of FeCN. Figure 4b shows the CV for the redox couple [Fe(CN)_6_]^3-/4-^of Au|MSA, the redox peaks of [Fe(CN)_6_]^3-/4- ^diminished and peak-to-peak separation increased when the pLys/GMPs-anti-p24 layers were modified on Au|MSA to form the immunosensor. In pH 6.5 PBS, CVs of Au|MSA/{pLys/GMPs-anti-p24}_2_ immunosensor, the experiment results indicated that nano GMPs, pLys, MSA, anti-p24 had no electrical activity, therefore the oxidation and reduction peaks correspond to the reversibility of the oxidation-reduction of FeCN. When the number of multilayers (n) increased, the immunosensor exhibits increased peak currents (see Figure 4c-e). The electrode response increased because the outer layer of pLys is positively charged and can easily absorb [Fe(CN)_6_]^3-/4-^ and GMPs have good conductivity, which can increase the electron transmission of [Fe(CN)_6_]^3-/4-^. Literature reports state that nano Au [21] and Fe_3_O_4 _particles[22] not only strengthen the electron transfer function of Au surfaces, but also supply active points for adsorbing antibodies,and our research finds that when nano Au and Fe_3_O_4_ form GMPs composites, these enhanced effects can be superimposed. These CVs are similar to those observed in poly-toluidine blue/nano-Aus electrode [20]. When more layers are added, the peak current increases.

**Figure 5:**
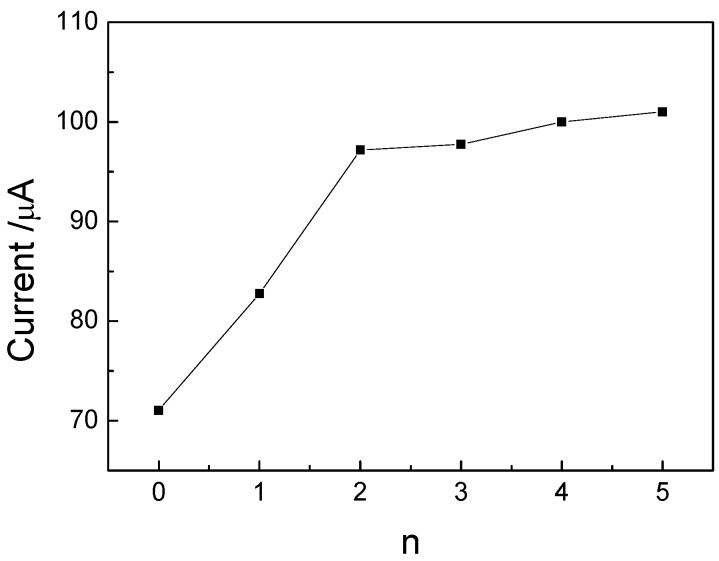
The comparison of peak current of the immunosensor coated with different numbers of layers.

A comparison of the current responses of immunosensors with different numbers of layers (n) is shown in Figure 5. For n ≥ 3, no increase of current was obvious. Because the preparation time for one layer is very time-consuming (7 h), two layers were chosen for fabricating the immunosensor. When the immunosensor was incubated in 10 ng/mL p24, the CV current diminished because the immuoreactant of anti-p24 and p24 was formed in the surface of the electrode, which is non-conductive and prevents the electron transformation of FeCN to the electrode. The cyclic voltammetry (CV) curves of the immunosensor at scan rates from 50 to 300 mV/s showed the potential divergence (ΔE_p_) between anodic and cathodic current peak increased with the change of scan rates, so we can obtain the average rate of transferred electron is 1.57 ± 0.12 s^-1^. The surface coverage on the surface of MSA was estimated to be 9.02 ± 1.13 × 10^-10^ mol/ cm^-2^, which is same as the sub-monomolecular value [13]. The number of electron transferred was obtained as n = 1.07 ≈ 1, corresponding to the redox of FeCN(Fe(IV)CN→Fe(III)CN). CVs of the immunosensor in pH 4.0–8.0 PBS showed the redox peak potential shifts negatively with the increase of pH. ∂E_pa_/∂pH = -52 mV/pH; ∂E_pc_/∂pH = -54 mV/pH. Due to the number of electrons transferred (z) of FeCN was 1, the number of H^+ ^involved in the reaction was 1.

#### 2.2.2. Electrochemical behaviors of the immunosensor and determination of p24

**Figure 6:**
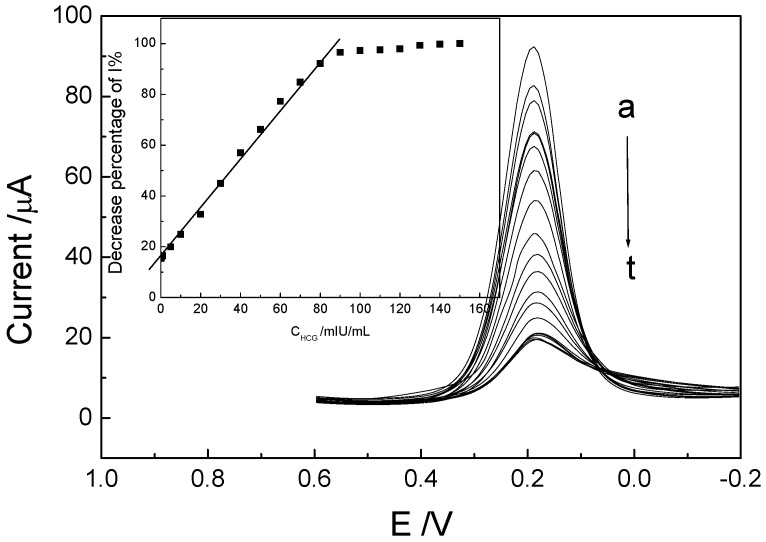
DPVs of the theimmunosensor incubated in p24 solution with different concentrations of a~t: 0, 0.1, 0.5, 1, 5, 10, 20, 30, 40, 50, 60, 70, 80, 90, 100, 110, 120, 130, 140, 150 ng/mL; the insert**: **ΔI/I*_H _*%~C_p24._

Differential pulse voltammetry (DPV) was employed for the determination of p24 due to its higher sensitivity than CVs. It can be seen that the peak current of FeCN by DPV decreased obviously when the immunosensor was incubated in p24 solution with different concentration (0~150 ng/mL) for 30 min (Figure 6a-t). And the ratio of decreased current value (ΔI) and I*_H_* (the highest I without p24) was proportional to the p24 concentration in the range from 0.1 to 100.0 ng/mL (Figure 6-insert). 

### 2.3. The optimum conditions of the immunoassay

The effect of the concentration of GMPs-anti-p24 used for fabrication of the immunosensor was also investigated when different concentration of anti GMPs-p24 was immersed in the solution containing 100 ng/mL p24 antibody at 25 °C from 0 to 7 h. The current declined, then essentially reached a stable value, representing the adsorption of antibody on GMPs-anti-p24 have reached saturation. Consequently the soaking time of 100 ng/mL GMPs-anti-p24 was 6 h. The performance of the immunosensor is usually related to the incubation temperature, the time, and the pH value of the detection solution. The dependence of the electrochemical behavior of the immunosensor on the pH of the working solution containing 1.0 mmol/L FeCN was studied. The peak current of the immunosensor increased with an increase of pH values ranged from 3.51 to 7.0, and the peaks became weak when the pH reached 6.6, thus the optimal pH value was 6.5. The amperometric response increased linearly with the FeCN concentration over the range from 0.1 to 1.0 mmol/L**, **and when the FeCN concentration was higher than 1.0 mmol/L, the ∆I tended to a constant value. Therefore, 1.0 mmol/L FeCN was chosen for the whole experiment. The effect of incubation time on the amperometric responses of the immunosensor was investigated. The value of ∆I_0 _increased with increasing incubation time and tended to a maximum value at 30 min, and at this time immune response on the electrode surface was completely finished. The average incubation time by the sensor was 20 min or so which shortened 3-fold compared with that of ELISA method which was more than 60 min [3]. This is because detection by the proposed method is based on a one-step immunoassay which reduced the need for addition of secondary antibody in the sandwich ELISA method.

### 2.4. Interference experiments, regeneration of the electrode surface and durability

Interference experiments were performed to assess whether the immunosensor could respond selectively to p24. The immunosensor was used to detect two 10 ng/mL p24 incubation solutions – one solution with an interferent [400 ng/mL α-fetoprotein (AFP); 2 µg/mL BSA, ascrobic acid (AA), uric acid (UA), dopamine (DA), L-lysine] and the other without. The results shown that the peak current responses in the two solutions showed less then 3.2% difference, which means that the immunosensor in this study could respond to p24 specifically. 

A small amount of serum protein could be adsorbed on the surface of electrode as the determination times increase, so that the regeneration of immunosensor is very important for their practical use. The prepared immunosensor could be regenerated by simply immersing it in a 0.1 mol/L guanidine hydrochloride-PBS solution for 12 h and removed to wash with water after each determination. A relative standard deviation (R.S.D.) of 2.6% was obtained when the electrode was used for eight consecutive measurements. The stability of the successive assays was studied. After 100 CV measurements in working buffer, an R.S.D. of 2.9% was obtained The long-time stability of the immunosensor was also investigated over a 75 day period. The immunosensor had acceptable storage stability with 94.3% of initial activity remaining after the 75 days storage periods at 4 °C. This result indicates that the immunosensor had acceptable storage stability and the present immunoassay method is suitable for the determination of p24 in human serum in routine clinical diagnosis.

### 2.5. Determination of p24 in real serum samples

We added 0.1~150 ng/mL p24 standards to normal human serum for a simulation in which the immunosensor was used to determine p24. The results are shown in Figure 7. The added p24 led to a linear decrease of the FeCN response current in p24 concentration ranges from 0.1~100 ng/mL with a detection limit of 0.05 ng/mL (3σ). Three real patient serum samples were determined by the method and the results compared with those obtained from a standard ELISA method, which were very close to standard results. The recovery was in the range from 94% to 103%, indicating that the immunosensor is suitable for the determination of HIV p24 in serum. The results are shown in Table 1.

**Table 1:** The determination of HIV p24 in real serum samples (n = 7).

Sample	HIV p24 (content)The concentration of HIV p24 (ng/mL)				
	Our method	ELISA method	RSD %	Added	Recovery %
1	2.3	2.5	2.7	2.0	102.2
2	5.4	5.2	2.5	5.0	92.6
3	2.2	2.3	2.6	2.0	94.3

## 3. Experimental

### 3.1. Reagents

HIV-1 p24 ELISA Kit (Cosmo Bio USA, Carlsbad, CA, USA): 5~500 ng/mL p24 and 100 ng/mL anti-p24 antibody. The p24 and anti-p24 solution were stored in the frozen state, and standard solutions were prepared with double distilled water. Bovine serum albumin (BSA, 96–99%), sulfanilic acid (SAA, Fluka Co.USA), mercaptosuccinic acid (HOOCCH_2_CH(SH)COOH, 98%, Merck), poly(L-lysine) hydrobromide (pLys, MW 67,900 g mol-1, Sigma Aldrich), GMP nanoparticles with diameter between 30 to 50 nm (Beimei Bio-Co. Shanxi Province, China), Human Serum Albumin (HSA, 96–99%) (Sigma, USA) were obtained commercially. All chemicals and solvents used were of analytical grade and were used as received. All reagents were brought to room temperature before usage. Distilled water was used for all experiments. HIV infected patients’ serum was provided by NanFang Medical University (three male samples, 2009-5-3). 

### 3.2. Apparatus

Cyclic voltammetric measurements (CV), differential pulse voltammetry (DPV) and electrochemical impedance spectroscopy (EIS) were carried out with a CHI 600 B electrochemistry workstation (Shanghai CH Instruments Co., China). A three-compartment electrochemical cell contained a platinum wire auxiliary electrode, a saturated calomel reference electrode (SCE) and the modified electrode as working electrode. The size of the GMP film was estimated from scanning electron microscopy (SEM; Hitachi S-3400N spectrometer, Japan). The sizes of GMPs, GMP-anti-p24 and pLys/GMPs-anti-p24 membrane were characterized by transmission electron microscopy (TEM; TECNAI 10, Philips, Holland). The elemental composition of GMPs was characterized by X-ray fluorescence spectrometry (S2 X-RANGER, Bruker Co., Germany)

### 3.3. Preparation of GMPs-anti-p24

The purified GMPs with diameter of 100 nm were dried under vacuum for 24 h at 50 °C. Afterwards, GMP nanoparticles (10 mg) were treated in toluene solution for 6 h at room temperature with slightly stirring, and then purified by centrifugation and redispersion in pH 6.5 PBS. The purified GMPs were incubated in anti-p24 antibody solution (100 ng/mL) at 4 °C for 12 h with light stirring. The excess anti-p24 antibody in supernatant was removed using a magnetic field and rinsed with PBS (pH 6.5). Following that, the anti-p24 antibody-modified GMPs were treated with 3.0 mg/mL BSA–PBS at 37 °C for 1 h to block the unreacted and nonspecific sites of GMPs. Finally, the synthesized bionanoparticles were stored at 4°C when not in use.

### 3.4. Fabrication of the immunosensor of Au|MSA/{pLys/GMPs-anti-p24}n

**Scheme 1:**
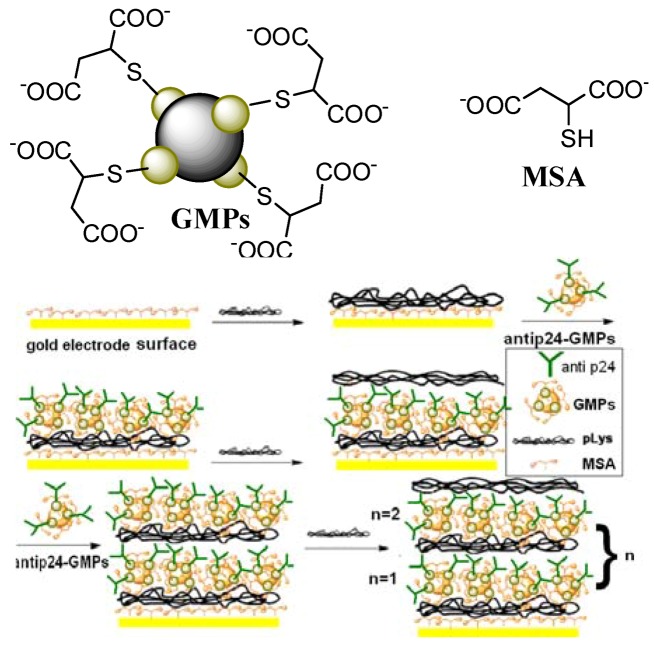
Schematic illustration of the p24 immunosensor assembly.

This procedure was performed according to the literature [19]. Freshly cleaned gold electrodes were immersed in a toluene solution (10 mol/L) of MSA for 18 h, and then the carboxylate groups of the acid adsorbed on the surface were activated to show negative charge by immersion in basic solution (0.2 mol/L NaOH) for 10 min, followed by washing with Millipore water. The modified electrode (Au|MSA) was then immersed in PBS solution of pLys (1 mg/mL) for 1 h. The positively charged polymer was then adsorbed on the negatively charged MSA membrane of Au|MSA with electrostatic force to form the Au|MSA/pLys electrode. Then Au|MSA/pLys was immersed in GMPs-anti-p24 anions for 6 h. The concentration of the GMPs solution was 0.22 mg/mL. After every layer deposition, the electrodes were washed with Millipore water to remove unbound polymer or GMPs. Alternating layers (Scheme 1) of cationic pLys and anionic GMPs were deposited up to maximum *n* = 2 layers on the electrode surface. The number of layers deposited is limited by the electrode response. The buildup of multilayers for the spectroscopic measurements was performed on quartz slides coated with 3-aminopropyltriethoxysilane (3APTS) proceeding as described in the literature [18,19]. The procedures used for construction of the immunosensor were shown in Figure 1. The experiments were always carried out under an atmosphere of N_2._

### 3.5. Experimental measurements

The analytical procedure for the immunoassay was based on the inhibition of immunocomplex formation by electron transfer between FeCN and the electrode. According to the literature [7] we determine the amperometric response *I_H_* of the immunosensor electrolyzed for 120 s at a potential of 200 mV in 5 mL anaerobic pH 6.5 PBS in 0.1 mol/L FeCN, then the immunosensor was incubated with p24 solution at 37 °C for 30 min, and amperometric response *I* in the same buffer with FeCN determined under the same conditions. The percentage decrease of the amperometric response of the immunosensor after incubation is given by the following expression: (*I_H_- I)/ I_H _*× 100%. The ratio is proportional to p24 concentration in a certain range. The ELISA tests were performed as follows: 1) each well of antibody coated plate is washed twice with 20-fold diluted wash buffer (350 μL); 2) diluted antigen standard (0, 7.5, 15, 30, 60 ng/mL) or specimen solutions (200 μL) are added to the washed wells, and incubated at 37 °C for 30 min; 3) the solutions in the wells are removed by aspiration and the wells washed three times with 20-fold diluted wash buffer (350 μL); 4) diluted biotinyl antibody (100 μL) is added to the washed wells and incubated at 37 °C for 30 min; 5) the solutions in the wells are removed by aspiration and the wells washed three times with 20-fold diluted wash buffer (350 μL); 6) diluted Enzyme-Labeled p24 antibody (100 μL) was added into the washed wells, and incubated at 37 °C for 30 min; 7) the solutions in the wells are removed by aspiration and the wells washed three times with 20-fold diluted wash buffer (350 μL); 8) substrate solution (100 μL) was added into the washed wells and incubated at room temperature for 30 min; 9) after washing four times in 0.1mol/L (pH6.5) phosphate buffer saluting (PBS), the color was developed with tetramethylbenzidine (TMB) for exactly 5 min and the optical density (OD) was read at 630 nm. The assays conducted during the development phase were performed in triplicate and the assays performed to test the patient samples were performed in duplicate. The average OD_630_ of duplicate or triplicate wells was plotted against the dilution factor for each test specimen on the same graphs with positive and negative serum specimens.

Electrochemical impedance tests: The modification procedures were monitored using electrochemical impedance spectroscopy (EIS) in the solution of 0.1 mol/L KCl and 5.0 mmol/L Fe(CN)_6_^3/4-^ at room temperature. A three-electrode system was used for recording the impedance spectra. Au|MSA/{pLys/GMPs-anti-p24}_n_ served as the working electrode. A platinum electrode and saturated calomel electrode (SCE) were used as the auxiliary and the reference electrode, respectively.

## 4. Conclusions

A stable, sensitive and separation-free amperometric immunosensor for rapid determination of HIV p24 in human serum has been successfully fabricated based on the electrostatic interaction between oppositely charged molecules and nanometer-sized Fe_3_O_4_(core)/Au colloidal(shell) particles (GMPs). Due to the strong electrostatic force of attraction between negatively charged GMPs and positively charged pLys, the p24 antibody coated nano GMPs anions can be easily absorbed on cationic polymers-pLys to form multilayer films on the electrode by a layer-by-layer assembly process. GMPs were used to provide a larger electrode surface area and an easier antibody immobilization; the mediator attached on the electrode surface simplified the experimental design and reduced the consumption of expensive reagents. The modified process was simple, and the results have good reproducibility. This amperometric immunosensor is very suitable for the detection of p24 in the "window period", and has potential application value for the early diagnosis of HIV. The immunosensor shows high sensitivity, low detection limit, and satisfactory storage stability which should be useful for the practical diagnosis of HIV.

## References

[1] Zhang K., Ma S.H. (2002). Epidemiology of HIV in China. Brit. Med. J..

[2] Albert J., Fenyo E.M. (1990). Simple, sensitive, and specific detection of human immunodeficiency virus type 1 in clinical specimens by polymerase chain reaction with nested primers. J. Clin. Microbiol..

[3] Gurtler L., Muhlbacher A., Michl U., Hofmann H., Paggi G., Bossi V., Thorstensson R., Villaescusa R.G., Eiras A., Hernandez J., Melchior W., Donie F., Weber B. (1998). Reduction of the diagnostic window with a new combined p24 antigen and human immunodeficiency virus antibody screening assay. J. Virol. Meth..

[4] Sickinger E., Stieler M., Kaufman B., Kapprell H., West D., Sandridge A., Devare S., Schochetman G., Hunt J.C., Daghfal D. (2004). Multicenter evaluation of a new, automated enzyme-linked immunoassay for detection of human immunodeficiency virus-specific antibodies and antigen. J. Clin. Microbiol..

[5] Sutthent R., Gaudart N., Chokpaibulkit K., Tanliang N., Kanoksinsombath C., Chaisilwatana P. (2003). p24 antigen detection assay modified with a booster step for diagnosis and monitoring of human immunodeficiency virus type 1 infection. J. Clin. Microbiol..

[6] Weber B., Gurtler L., Thostensson R., Michl U., Mulbacher A., Burgisser P., Vilaesscusa R., Eiras A., Gabriel C., Stekel H., Tanprasert S., Oota S., Silvestre M., Marques C., Ladeira M., Rabenau H., Berger A., Schmitt U., Melchior W. (2002). Multicenter evaluation of a new automated fourth-generation human immunodeficiency virus screening assay with a sensitive antigen detection module and high specificity. J. Clin. Microbiol..

[7] Wu J., Tang J.H., Dai Z., Yan F., Ju H.X., Murr N.E. (2006). A disposable electrochemical immunosensor for flow injection immunoassay of carcinoembryonic antigen. Biosens Bioelectron.

[8] He X.L., Yuan R., Chai Y.Q., Shi Y.T. (2008). A sensitive amperometricimmunosensor for carcinoembryonic antigen detection with porous nanogold film and nano-Au/chitosan composite as immobilization matrix. J. Biochem. Biophys. Methods.

[9] Thevenot D.R., Toth K., Durst R.A., Wilson G.S. (2001). Electrochemical biosensors: recommended definitions and classification. Biosens. Bioelectron..

[10] Wu L., Zhang X.J., Ju H.X. (2007). Amperometric glucose sensor based on catalytic reduction of dissolved oxygen at soluble carbon nanofiber. Biosen. Bioelectron..

[11] Wang J. (2005). Nanomaterial-based electrochemical biosensors. Analyst.

[12] Baron R., Willner B., Willner I. (2007). Biomolecule-nanoparticle hybrids as functional units for nanobiotechnology. Chem. Commun..

[13] Wang J. (2007). Nanoparticle-based electrochemical bioassays of proteins. Electroanalysis.

[14] Guo S., Wang E. (2007). Synthesis and electrochemical applications of gold nanoparticles. Anal. Chim. Acta.

[15] Kim J.H., Hwang J.H., Lim T.Y. (2009). A layer-by-layer self-assembly method for organic-inorganic hybrid multilayer thin films. J. Ceram. Process. Res..

[16] Ban Z., Barnaov Y.A., Li F., Golup V.O., O’Conner C.J. (2005). The synthesis of core shell iron and gold nanoparticles and their characterization. J. Mater. Chem..

[17] Gupta A.K., Gupta M. (2005). Synthesis and surface engineering of iron oxide nanoparticles for biomedical applications. Biomaterials.

[18] Constantine C.A., Mello S.V., Dupont A., Cao X.H., Santos D.J., Oliveira O.N.J., Strixino F.T., Pereira E.C., Cheng T.C., Defrank J.J., Leblance R.M. (2003). Layer-by-layer self-assembled chitosan/ poly (thiophene-3-acetic acid) and organophosphorus hydrolase multilayers. J. Am. Chem. Soc..

[19] Mariana C., Vladimir G.M., Manzanares J.A., Carlos P., Rubin G., Fernando S. (2005). Electrochemical Characterization of Polyelectrolyte/Gold NanoparticleMultilayers Self-Assembled on Gold Electrodes. J. Phys. Chem. B..

[20] Li X.L., Yuan R., Chai Y.Q., Zhang L.Y., Zhuo Y., Zhang Y. (2006). Amperometric immunosensor based on toluidine blue/nano-Au through electrostatic interaction for determination of carcinoembryonic antigen. J. Biotechnol..

[21] Wu Y., Zheng J.W., Li Z., Zhao Y.R., Zhang Y. (2009). A novel reagentless amperometric immunosensor based on gold nanoparticles/TMB/Nafion-modified electrode. Biosens. Bioelectron..

[22] Wang S.F., Tan Y.M., Zhao D.M., Liu G.D. (2008). Amperometric tyrosinase biosensor based on Fe_3_O_4_ nanoparticles-chitosan nanocomposite. Biosens. Bioelectron..

[23] Chai R., Yuan R., Chai Y.Q., Ou C.F., Cao S.R., Li X.L. (2008). Amperometric immunosensors based on layer-by-layer assembly of gold nanoparticles and methylene blue on thiourea modified glassy carbon electrode for determination of human chorionic gonadotrophin. Talanta.

